# Bacteria Associated with Spores of Arbuscular Mycorrhizal Fungi Improve the Effectiveness of Fungal Inocula for Red Raspberry Biotization

**DOI:** 10.1007/s00248-024-02364-5

**Published:** 2024-03-11

**Authors:** Rafał Ważny, Roman J. Jędrzejczyk, Piotr Rozpądek, Agnieszka Domka, Krzysztof M. Tokarz, Martyna Janicka, Katarzyna Turnau

**Affiliations:** 1 Małopolska Centre of Biotechnology, Jagiellonian University in Kraków, Gronostajowa 7a, 30-387 Kraków, Poland; 2 W. Szafer Institute of Botany Polish Academy of Sciences, Lubicz 46, 31-512 Kraków, Poland; 3 Department of Botany, Physiology and Plant Protection, University of Agriculture in Krakow, Aleja Mickiewicza 21, 31‐120, Kraków, Poland; 4 Department of Nanobiology and Biomaterials, Military Institute of Hygiene and Epidemiology, Kozielska 4, 01-163 Warsaw, Poland; 5 Division of Microbiology, Department of Preclinical Sciences, Institute of Veterinary Medicine, Warsaw University of Life Sciences, Ciszewskiego 8, 02-786 Warsaw, Poland; 6 Institute of Environmental Sciences, Jagiellonian University in Kraków, Gronostajowa 7, 30-387 Kraków, Poland

**Keywords:** Biotization, AMF, Bacteria associated with AMF spores, Red raspberry, *Rubus idaeus*

## Abstract

Intensive crop production leads to the disruption of the symbiosis between plants and their associated microorganisms, resulting in suboptimal plant productivity and lower yield quality. Therefore, it is necessary to improve existing methods and explore modern, environmentally friendly approaches to crop production. One of these methods is biotization, which involves the inoculation of plants with appropriately selected symbiotic microorganisms which play a beneficial role in plant adaptation to the environment. In this study, we tested the possibility of using a multi-microorganismal inoculum composed of arbuscular mycorrhizal fungi (AMF) and AMF spore-associated bacteria for biotization of the red raspberry. Bacteria were isolated from the spores of AMF, and their plant growth-promoting properties were tested. AMF inocula were supplemented with selected bacterial strains to investigate their effect on the growth and vitality of the raspberry. The investigations were carried out in the laboratory and on a semi-industrial scale in a polytunnel where commercial production of seedlings is carried out. In the semi-industrial experiment, we tested the growth parameters of plants and physiological response of the plant to temporary water shortage. We isolated over fifty strains of bacteria associated with spores of AMF. Only part of them showed plant growth-promoting properties, and six of these (belonging to the *Paenibacillus* genus) were used for the inoculum. AMF inoculation and co-inoculation of AMF and bacteria isolated from AMF spores improved plant growth and vitality in both experimental setups. Plant dry weight was improved by 70%, and selected chlorophyll fluorescence parameters (the contribution of light to primary photochemistry and fraction of reaction centre chlorophyll per chlorophyll of the antennae) were increased. The inoculum improved carbon assimilation, photosynthetic rate, stomatal conductance and transpiration after temporary water shortage. Raspberry biotization with AMF and bacteria associated with spores has potential applications in horticulture where ecological methods based on plant microorganism interaction are in demand.

## Introduction

Plant-associated microorganisms play a decisive role in plant growth (biomass production) and stress tolerance [15, 17, 58]. In the last decades, intensive agriculture has significantly impacted the community structure of soil microorganisms which forms a natural reservoir of microorganisms for the plant. Thus, in modern agriculture, more attention is being paid to plant biotization with microorganisms that are capable of increasing mineral uptake by plants (as biofertilisers), fine-tunning plant metabolism (as bioregulators) and resistance to abiotic and biotic stress (as bioprotectors) and thus play a beneficial role in plant adaptation to the environment [11, 22, 25].

Arbuscular mycorrhizal fungi (AMF) are commonly known to provide many benefits to the plant. AMF improve growth and nutritional quality of crops [4, 48] and protect plants against environmental stress such as pathogens and abiotic environmental conditions [40, 56]. Widespread occurrence of mycorrhizal symbiosis and the obligatory nature of this plant (fungus association for many plant species) have long directed the attention of the scientific community to the practical use of mycorrhiza in protection of endangered plants, in phytostabilization of toxic wastes and in sustainable agriculture to improve food security [8, 39, 54].

In recent years, more attention has been paid to the use of multi-microorganismal inocula that include AMF and other groups of symbiotic organisms [7]. This approach provides the plant with microorganisms possessing a variety of plant growth-promoting traits. It has been demonstrated that AMF can be used effectively in co-inoculation mainly with endophytic fungi, endophytic bacteria and soil bacteria [7, 18, 56]. The effects of such practises differ in between studies. Plant growth was affected either positively or negatively compared to the inoculation with a single microorganism. This is quite understandable since successful biotization relies on appropriately selected microorganisms used for inoculation of a given plant cultivar. Mutual interactions between the microorganisms and the plant host seem to determine the efficiency of plant growth promotion [51].

Bacteria associated with AMF spores have been recently investigated on several occasions [1, 3, 9, 16, 29, 41, 53] and represent different taxa including Proteobacteria (*Achromobacter, Agrobacterium, Aquitalea, Bosea, Burkholderia, Cellvibrio, Cupriavidus, Desulfovibrio, Duganella, Ensifer, Enterobacter, Herbaspirillum, Ideonella, Lysobacter, Massilia, Methylibium, Mitsuaria, Proteus, Pseudomonas, Ralstonia, Rheinheimera, Rhizobium, Sinorhizobium*), Actinobacteria (*Amycolatopsis, Arthrobacter, Curtobacterium, Gordonia, Leifsonia, Mycobacterium, Nocardia, Propionibacterium, Streptomyces*) Firmicutes (*Bacillus, Brevibacillus, Paenibacillus*) and Bacteroidetes (*Flexibacter*). Although the first reports of bacteria in AMF spores date back to over 50 years ago [31, 32], their role in plant-microorganism interactions is still poorly recognised. Bacteria associated with AMF spores, *Bacillus subtilis, Pseudomonas diminuta*, *Enterobacter hormaechei, Bacillus* sp., *Bacillus thuringiensis* and *Paenibacillus rhizosphaerae* have been shown to be able to inhibit the growth of pathogens and to activate the development of hyphae of *Gigaspora* [9, 16]. Functional analysis of 43 bacterial strains isolated from spores of *Gigaspora margarita* revealed that about 30% of them stimulated spore germination, nearly 60% solubilised phosphorus, 15% degraded chitin and three taxa, *Curtobacterium, Ensifer* and *Bacillus,* improved growth of alfalfa [29]. According to some authors, certain plant growth-promoting functions provided by AMF may be related to mycorrhiza-associated bacteria [16, 53]. Keeping in mind the multiple benefits provided to plants by AMF or AMF spore-associated bacteria, inocula based on both groups of microorganisms can be used for sustainable crop production.

The aims of this study were to test the possibility of improving the efficiency of AMF-based inocula for biotization of the red raspberry by supplementing different compositions of AMF with bacteria isolated from AMF spores. We investigated how these multi-microorganismal inocula affect (1) plant growth in laboratory and greenhouse conditions and (2) the physiological response of plant to temporary water shortage. As a model plant, the red raspberry (*Rubus idaeus* L.) was selected. This is an important crop species with growing consumer interest. Its fruits are desirable for their taste and nutritional properties, [10]. These properties result in a high demand for raspberry fruit worldwide.

## Materials and Methods

### Experimental Design

The investigations were carried out in three steps. In the first step, the bacterial components for the inoculum were isolated and selected based on the plant growth-promoting properties. In the second step, AMF inoculum supplemented with selected bacteria was used to inoculate raspberries in laboratory experiments. In the third step, the efficiency of the selected AMF and bacterial supplement for AMF was verified in semi-industrial scale raspberry production in a polytunnel.

### Isolation and Identification of Bacteria from Spores

Bacteria were isolated from the spores of arbuscular mycorrhizal fungi. Roots of plants were collected from raspberry (five plants) and blackberry (one plant) plantations and used for inoculum preparation in the pot (16 pots) culture system (in association with *Plantago lanceolata*) as described in Orłowska et al. [36]. Sixteen samples of air-dried rhizosphere (5 g) soil from trap cultures were used for AMF identification. The obtained spores were divided into groups according to their morphological features and subsequently identified according to their SSU rRNA sequence. AML1 (5′-ATCAACTTTGATGGTAGGATAGA-3′) and AML2 (5′-GAACCCAAACACTTTGGTTTC C-3′) [28] primers were used for nested PCR followed by a Strata Clone PCR Cloning Kit (Agilent, USA). Amplicons were purified and then sequenced (Sanger sequencing) bidirectionally by Macrogen Europe (The Netherlands). Sequences were compared with sequences available in the NCBI (National Centre for Biotechnology Information).

Spore-associated bacteria isolation was carried out from approximately 200 AMF spores. Spores were suspended in 1 mL sterile 0.9% NaCl in a 1.5-mL tube and shaken for 1 min using a vortex. Subsequently, spores were washed aseptically in 0.9% NaCl 15 times (centrifugation at 1500 × *g* for 1 min) [5] and crushed with a micro pestle. The suspension was heated at 80 °C for 10 min in order to isolate spore-forming bacteria. Then, 100 µL of suspension was plated in five replicates on five different media: TSB and NA (Tryptic Soy Broth and Nutrient Agar—for heterotrophic bacteria isolation), WAM (Waksman’s Agar Medium—aimed at actinobacteria), Winogradsky Culture Agar (N-free medium aimed at nitrogen-fixing bacteria) and minimal medium with chitin as carbon source (for chitinolytic bacteria). Plates were incubated at 28 °C in darkness for 7 days. Emerging bacteria were transferred into new media. Bacteria selected for the inoculum were identified based on the sequence of 16S rDNA region. DNA was isolated with a DNA Mini Kit (Syngen). 27F (5′-GAGTTTGATCCTGGCTCAG-3′) and 1492R (5′-GGTTACCTTGTTACGACTT-3′) primers were used for PCR [52]. Bacterial amplicons were sequenced and analysed as described for fungi.

### Plant Growth-Promoting Properties of Isolated Bacteria Selection for the Inoculum

#### Phosphate Solubilisation

Bacteria were examined to test their inorganic phosphate solubilising potential. Bacteria were cultivated on NA (Nutrient Agar) medium at 30 °C in darkness for 2 days. Subsequently, bacteria were cultured on Pikovskaya Agar medium [38] for 7 days at 30 °C in darkness (*N* = 3). Tri-calcium phosphate was the source of insoluble phosphate. Phosphate solubilising activity was indicated as a clearance around the microorganism colony.

#### Phytate Solubilisation

The ability of bacteria to solubilise organic phosphate was examined on Phytate Screening Medium (PSM; 10 g · L^−1^ d-glucose, 4 g · L^−1^ C_6_H_18_P_6_O_24_·12Na·H_2_O, 2 g · L^−1^ CaCl_2_, 5 g · L^−1^ NH_4_NO_3_, 0.5 g · L^−1^ KCl, 0.5 g · L^−1^ MgSO_4_·7H_2_O, 0.01 g · L^−1^ FeSO_4_·7H_2_O, 0.01 g · L^−1^ MnSO_4_·H_2_O, 15 g · L^−1^ agar, pH 7) for 7 days at 30 °C in darkness (*N* = 3). Organic phosphate solubilising activity was indicated as a clearance around the microorganism colony [5].

#### Production of Indole Acetic Acid (IAA)

Bacteria were cultured in Luria–Bertani Broth (LBB) supplemented with 1 mg · L^−1^ L-tryptophan (Sigma-Aldrich) at 20 °C at 200 rpm for 24 h and then centrifuged at 7500 × *g* for 10 min. The supernatant (1 mL) was mixed with 2 mL Salkowski reagent (1.2% FeCl_3_ in 37% sulphuric acid) in a well plate and incubated for 30 min in darkness (*N* = 3) [23]. The production of IAA was assessed based on colour development (‘-’ no colour development, no production,‘ ± ’ pink pale, low production; ‘ + ’ light pale, production; ‘ +  + ’ bright purple, moderate production; ‘ +  +  + ’ dark purple, high production).

#### Siderophore Production

To assess siderophore production, the modified blue agar chromeazurol S (CAS) method by Schwyn and Neilands [44] was used. Four different solutions (1–4) were prepared, mixed in the following order: 2, 3, 4, 1 and aseptically poured onto plates. Solution 1: 100 mL dd H_2_O, 2.7 g FeCl_3_ · 6H_2_O, 180 µL HCl (0.56 mM), 60.5 g Chromeazurol S (CAS) and 72.8 mg HDTMA bromide, autoclave; Solution 2: 800 mL dd H_2_O, 0.3 g KH_2_PO_4_, 0.5 g NaCl, 1 g NH_4_Cl, 30.24 g PIPES (to dissolve PIPES pH was adjusted to 6.8) and 15 g agar, autoclave; Solution 3: 70 mL dd H_2_O, 2 g glucose, 2 g mannitol, 0.493 g MgSO_4_ · 7H_2_O, 11 mg CaCl_2_ · 2H_2_O, 1.17 mg MnSO_4_ · H_2_O, 1.4 mg H_3_BO_4_, 0.04 mg CuSO_4_ · 5H_2_O, 1.2 mg ZnSO_4_ · 7H_2_O, 1 mg Na_2_MoO_4_ · 2H_2_O, autoclave; Solution 4: 3 g hydrate of casein was dissolved in 30 mL dd H_2_O and filter sterilised. Bacteria were cultured on CAS blue agar for 14 days (*N* = 3). Bacteria that possessed the ability to produce siderophores removed iron from the dye complex, and the medium colour changed from blue to orange.

### Plant Growth Response Tests

#### Laboratory Experiment

Raspberry plant cuttings were cultured in vitro in Murashige and Skoog Medium supplemented with 1.2 mg · L^−1^ α naphthalene acetic acid (NAA) and 0.3 mg · L^−1^ indole-3-butyric acid (IBA) for 4 weeks and subsequently transferred to a mixture of garden soil (supplied by ARO, Poland), sand and clay (in equal volumes) supplemented with 40 mL·L^−1^ rock phosphate (Siarkopol, Poland) in pot cultures [56]. Prior to planting, the soil was sterilised at 100 °C for 1 h for 3 consecutive days and sprayed with sterile water for 2 weeks. During transfer, plants were inoculated by adding 5 mL of AMF inoculum and 2 mL of bacterial inoculum to the planting hole. Plants were grown in a greenhouse under natural day/night conditions with additional light during the day (12 h), approximately 500 µmol m^−2^ · s^−1^, and were watered alternately with tap water and nutrient solution (Long Ashton) every 2 to 3 days to keep the substrate humidity at the level of approximately 60%. Plants were subject to four different treatments (with 20 replicates per treatment): (1) inoculation with AMF, (2) inoculation with bacteria, (3) inoculation with AMF + bacteria and (4) no-inoculation (control, without any supplementation). Three-month-old plants were transferred to bigger pots (volume 1 L). Plants were harvested after 5 months.

The AMF inoculum was a mixture of *Entrophospora lamellosa* (Dalpé, Koske & Tews) Błaszk., Niezgoda, B.T. Goto & Magurno*, Entrophospora* sp. R.N. Ames & R.W. Schneid. 1979 and *Rhizophagus irregularis* (Błaszk., Wubet, Renker & Buscot) C. Walker & A. Schüßler prepared separately in pot cultures of *Plantago lanceolata* L and mixed (v:v:v; 1:3:3). Approximately 5 mL of the inoculum, containing spores, mycelium and colonised root fragments was mixed with the upper layer of substrates.

The bacterial inoculum was a mixture of bacteria isolated from spores of AMF: *Paenibacillus amylolyticus* (Nakamura 1984) Ash et al. 1994, *P. contaminans* Chou et al. 2009*, P. alginolyticus* (Nakamura 1987) Shida et al. 1997*, Paenibacillus soli* Park et al. 2007*, Paenibacillus* sp. 1 Ash et al. 1994, *Paenibacillus* sp. 2 and one bacterial strain not associated with AMF spores, *Stenotrophomonas* sp. Palleroni and Bradbury 1993, from culture collection in the Institute of Environmental Sciences of the Jagiellonian University. Bacteria were cultured in TSB (Tryptone Soy Broth) medium in natural day/night cycles at 120 rpm at 30 °C for 96 h. Cultures were washed twice with sterile 0.9% NaCl (5000 g, 5 min) suspended in 50 mL 0.9% NaCl and mixed.

#### Tunnel Experiment

Raspberry plants were cultured in vitro for 4 weeks and transferred to garden soil (Novarbo) in pot culture (pot volume 40 mL). Plants were grown in a phytotron room for 6 weeks and then transferred to bigger pots (pot volume 200 mL) with a new substrate. The substrate was a mixture of garden soil (supplied by ARO, Poland), sand and clay (in equal volumes) supplemented with 40 mL·L^−1^ powdered rock phosphate (Siarkopol, Poland) [56]. The substrate was sterilised in 100 °C for 1 h for 3 consecutive days and sprayed with sterile water for 2 weeks. During transfer, plants were inoculated by adding 5 mL of AMF inoculum and 2 mL of liquid bacterial inoculum to the planting hole. Plants were grown in greenhouse under natural day/night conditions and were watered with tap water every 2 days. Plants were inoculated in June (*N* = 70) and harvested in September.

The inoculum used for raspberry biotization was a mixture of mycorrhizal fungi and bacteria associated with AMF spores. Mycorrhizal inoculum was a combination of four different AMF inocula from the collection of Institute of Environmental Sciences at Jagiellonian University in Kraków:

1. Rhizoglomus intraradices,
2. *Funneliformis mosseae* (T.H. Nicolson & Gerd.) C. Walker & A. Schüßler,
3. Mix3 (was composed of *R. intraradices, F. mosseae, F. constrictus* (Trappe) C. Walker & A. Schüßler*, F. geosporus* (T.H. Nicolson & Gerd.) C. Walker & A. Schüßler (1:1:1:1)) and
4. Mix4 (composed of R. intraradices, F. mosseae, F. constrictus, F. geosporus (4:3:1:1)).

Each of the four inocula was prepared separately in pot cultures of *Plantago lanceolata* L. and mixed (v:v:v:v, 2:2:1:1). Approximately 5 mL of the inoculum, containing spores, mycelium and colonised root fragments was mixed with the upper layer of the soil. The same bacterial inoculum as described in the “Laboratory Experiment” section was used.

### Photosynthetic Efficiency

Photosynthetic efficiency was determined as described in Strasser et al. [46]. Briefly, chlorophyll fluorescence measurements were performed with a Handy Pea fluorimeter (Hansatech Instruments, UK). One mature leaf from each plant (10 replicates) was dark-adapted for 20 min in special clips before the measurement. Data were processed with BIOLYZER software (Laboratory of Bioenergetics, Geneva, Switzerland).

### Gas Exchange in Response to Temporary Water Shortage

To apply water deficit, irrigation of cultures was discontinued for 7 days until the appearance of first water deficit symptoms in control plants (decrease in leaf turgor). Subsequently, plants were watered every 2 days. Two weeks later, the photosynthetic rate, stomatal conductance of H_2_O and transpiration rate were measured using LCpro-SD (ADC BioScientific Ltd., Hoddesdon, UK). All measurements were performed on the second leaf of randomly selected plants using a 6.25 cm^2^ chamber equipped with a mixed Red/Blue LED Light Source Head. The measurements were carried out under the following conditions: CO_2_ saturated conditions (600 µmol · mol^−1^ air), irradiance of 100 µmol (photons) · m^−2^ · s^−1^ red light intensity and a leaf temperature of 24 °C ± 0.5 °C in five biological replications for each group.

### Statistical Analysis

Statistical analysis was performed using Statistica 13 (Tibco). Differences between experimental groups were considered significant at *p* ≤ 0.05. Data normality and variance homogeneity were assessed with Shapiro–Wilk’s and Levene’s tests, respectively. Differences were tested using a *t*-test or analysis of variance (ANOVA) followed by Tukey’s post hoc tests.

## Results

### Selection of Bacteria for Inoculum Components

Fifty-six bacterial strains were isolated from AMF spores collected from the roots and rhizosphere of raspberry and blackberry plants. AMF spore morphotypes were identified as three different taxa: *Entrophospora lamellosa, Entrophospora sp.* and *Rhizophagus irregularis *(Table 1). Seventeen bacterial strains were isolated on TSA, 16 – on NA, 9 – on N-free medium, 9 – on WAM and 5 – on MM. 64% of the strains exhibited phytate solubilisation, whereas the ability to solubilise phosphate was shown in only 7% of the strains. Twenty-nine percent of strains were able to synthesise IAA and 20% to produce siderophores (Table 2). The strains that exhibited the highest rate of IAA production, phosphate and phytate solubilisation and siderophore production were selected for the inoculum (Table 1). Additionally, the bacterial strain *Stenotrophomonas* sp. from the culture collection at the Institute of Environmental Sciences of Jagiellonian University were included in the inoculum.

**Table 1:** Molecular identification of AMF and bacteria strains

Strain UNIJAG.PL	NCBI number	Identification	Reference sequence NCBI	Similarity
Arbuscular mycorrhizal fungi				
1951BAA005	OR961069	*Rhizophagus irregularis*	*Glomus irregulare FJ009618.1*	800/800 (100%)
1951BAA011	OR961070	*Entrophospora lamellosa*	*Glomus lamellosum AJ276087.2*	750/750 (100%)
1951BAA001	OR961067	*Entrophospora sp.*	*Entrophospora etunicata* MT626044.1	389/389 (100%)
			*Entrophospora lamellosa* KX879068.1	389/389 (100%)
1951BAA013	OR961071	*Rhizophagus irregularis*	*Glomus irregulare FJ009618.1*	745/745 (100%)
1951BAA015	OR961072	*Rhizophagus irregularis*	*Rhizophagus irregularis* CP110711.1	736/736 (100%)
1951BAA017	OR961073	*Entrophospora sp.*	*Claroideoglomus lamellosum FR773152.1*	750/750 (100%)
			*Glomus etunicatum AJ852598.1*	750/750 (100%)
1951BAA003	OR961068	*Entrophospora sp.*	*Entrophospora etunicata* MN726592.1	643/643 (100%)
			*Entrophospora lamellosa* MW642179.1	643/643 (100%)
742	OR961066	*Entrophospora sp.*	*Claroideoglomus lamellosum FR750221.1*	720/720 (100%)
			*Glomus etunicatum AJ852598.1*	720/720 (100%)
Bacterial strains isolated from AMF spores				
733.6 M	OR960747	*Paenibacillus amylolyticus*	*Paenibacillus amylolyticus* AB115960.1	938/938 (100%)
734.10 M	OR960748	*Paenibacillus soli*	*Paenibacillus soli* JQ342903.1	897/898 (99%)
735.19 M	OR960749	*Paenibacillus contaminans*	*Paenibacillus contaminans* NR_044325.1	700/700 (100%)
736.24 M	OR960750	*Paenibacillus alginolyticus*	*Paenibacillus alginolyticus* HQ236042.1	903/903 (100%)
737.30 M	OR960751	*Paenibacillus* sp.1	*Paenibacillus cineris* LN890143.1	939/939 (100%)
			*Paenibacillus favisporus* JN867753.1	939/939 (100%)
			*Paenibacillus rhizosphaerae* GU830879.1	939/939 (100%)
738.52 M	OR960752	*Paenibacillus* sp.2	*Paenibacillus pabuli* FJ189794.1	940/940 (100%)
			*Paenibacillus xylanilyticus* NR_029109.1	940/940 (100%)

**Table 2:** Characterisation of plant growth-promoting traits

Bacteria strain	Medium	Phosph. solub.^a^	Phytates solub.^a^	IAA produc.^b^	Sideroph. prod.^a^		Bacteria strain	Medium	Phosph. solub.^a^	Phytates solub.^a^	IAA produc.^b^	Sideroph. prod.^a^
1 M	TSA	+	+				29 M	WAM			+	
2 M	TSA						**30 M**	**WAM**		** + **	** + **	
3 M	TSA						31 J	WAM				
4 M	TSA	+	+				32 J	WAM			+ + +	+
5 M	TSA			+			33 J	WAM		+		
**6 M**	**TSA**	** + **	** + **				34 J	WAM		+		
7 M	TSA		+				35 J	WAM		+		+
8 M	TSA	+	+				36 J	TSA				
9 M	TSA		+				37 J	TSA		+		+
**10 M**	**TSA**		** + **	** + + + **			38 J	TSA			+ +	
11 M	TSA				+		39 J	TSA		+		
12 M	NA		+				40 J	NA				
13 M	NA			+ + +			41 J	NA				
14 M	NA		+				42 J	NA		+		
15 M	NA				+		43 J	NA		+		
16 M	NA			+ + +			44 J	WAM		+	+ +	+
17 M	NA						45 J	TSA		+	+ +	
18 M	NA						46 J	TSA		+		
**19 M**	**NA**		** + **	** + **	** + **		47 J	NA		+		
20 M	NA		+				48 J	N free		+		
21 M	NA						49 J	N free		+	+	
22 M	NA		+		+		50 J	N free		+		
23 M	N free		+				51 J	N free				
**24 M**	**N free**		** + **	** + **	** + **		**52 M**	**MM**		** + **		
25 M	N free		+				53 M	MM		+		
26 M	N free		+				54 J	MM		+	+ + +	
27 M	N free		+				55 J	MM				
28 M	WAM			+ +	+		56 J	MM		+	+ + +	+

^a^ + indicates the ability of phosphate/phytate solubilisation or siderophore production

^b^the production of IAA was classified based on colour development (‘ + ’ light pale, production; ‘ +  + ’ bright purple, moderate production; ‘ +  +  + ’ dark purple, high production)

Strains presented in bold were included in the inoculum

### Bacteria Isolated from AMF Spores Improve AMF-Inoculated Raspberry Growth

**Fig. 1:**
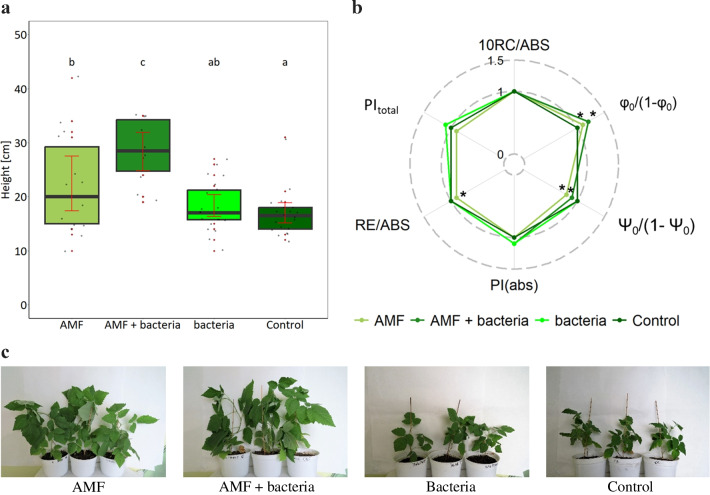
Height (**a**) and PSII efficiency of plants cultured in the greenhouse—laboratory experiment (JIP-test parameters: PIabs—absorbance performance index, PItotal—total performance index, Φ0/(1-Φ0)—contribution of light reactions for primary photochemistry, RC/ABS—fraction of reaction centre chlorophyll per chlorophyll of the antennae, Ψ0/(1-Ψ0)—electron transport beyond primary quinone acceptor and RE/ABS—contribution of the reduction of end equivalents are presented relative to entirely non-inoculated plants; statistically significant differences between particular treatments and those entirely non-inoculated plants are indicated by asterisk (*t*-test, *P* ≤ 0.05, *N* = 10) (**b**) and photographs (**c**) of red raspberry inoculated with AMF, AMF spore-associated bacteria. For each treatment, 25 seedlings were inoculated. Plants were grown in peat and perlite (5:1, v:v) and irrigated with water. Plants were harvested for analysis at the end of the growing season

Non-inoculated raspberry yielded 16.7 cm in height after 5 months of growth in the laboratory (Fig. 1a). Plants inoculated with AMF spore-associated bacteria were not significantly higher (18.2 cm) than control plants. Inoculation with AMF significantly increased plant height. Plants inoculated with the fungi yielded 22.1 cm. Co-inoculation with bacteria and AMF had the best effect on plant height. The plants yielded 28.4 cm which was significantly better in relation to control plants and plants inoculated with single: AMF or bacteria (Fig. 1a, c).

Plant vitality was assessed based on fluorescence of chlorophyll *a*. Out of the analysed parameters describing the efficiency of electron transport in PSII (and energy production), only two were significantly changed in inoculated plants in comparison to control. The contribution of light to primary photochemistry (Φ_0_/(1-Φ_0_)) was significantly higher in AMF-inoculated and AMF-bacteria co-inoculated plants compared to non-inoculated plants (Fig. 1b). However, electron transport beyond primary quinone acceptor (Ψ_0_/(1-Ψ_0_)) in these plants was significantly lower than in non-inoculated plants (Fig. 1b).

### Multi-microorganismal Inoculum Improves Plant Growth and Response to Temporary Water Shortage on a Semi-industrial Scale

**Fig. 2:**
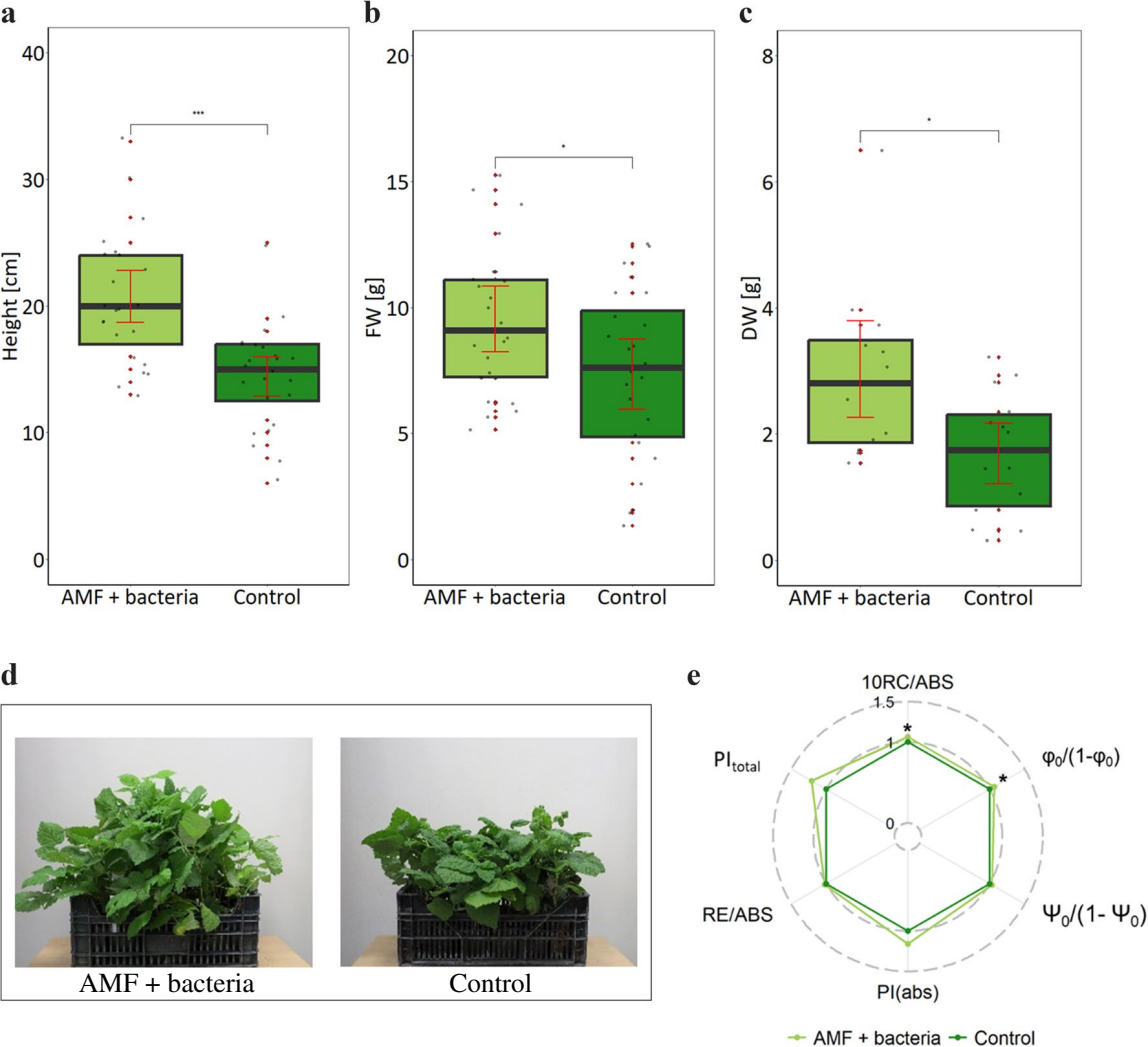
Parameters of plants from semi-industrial scale experiment: **a** height, **b** fresh weight, **c** dry weight, **d** photographic visualisation, **e** PSII efficiency (JIP-test parameters are described in Fig. 1) of red raspberry co-inoculated with AMF and AMF spore-associated bacteria. For each treatment, 70 seedlings were grown in greenhouse from June to September. Statistically significant differences between inoculated and non-inoculated plants are indicated by asterisk (*t*-test, *P* ≤ 0.05, *N* = 20)

The experimental setup in the semi-industrial scale experiment was simplified compared to the initial screening in the laboratory. In this experiment, we compared the growth of plants either supplemented with AMF and bacteria or not inoculated. At the end of the growing season, non-inoculated plants reached 14.4 cm in height. Inoculated plants were significantly higher than control plants yielding 20.6 cm (40% increase) (Fig. 2a). Plant inoculation increased plant fresh biomass (28% increase) and plant dry biomass (76% increase) (Fig. 2b–d).

Inoculation was beneficial for plant vitality. Two parameters describing fluorescence of chlorophyll, the contribution of light to primary photochemistry and fraction of reaction centre chlorophyll per chlorophyll of the antennae were significantly higher in AMF-bacteria co-inoculated plants than for non-inoculated plants (Fig. 2e).

**Fig. 3:**
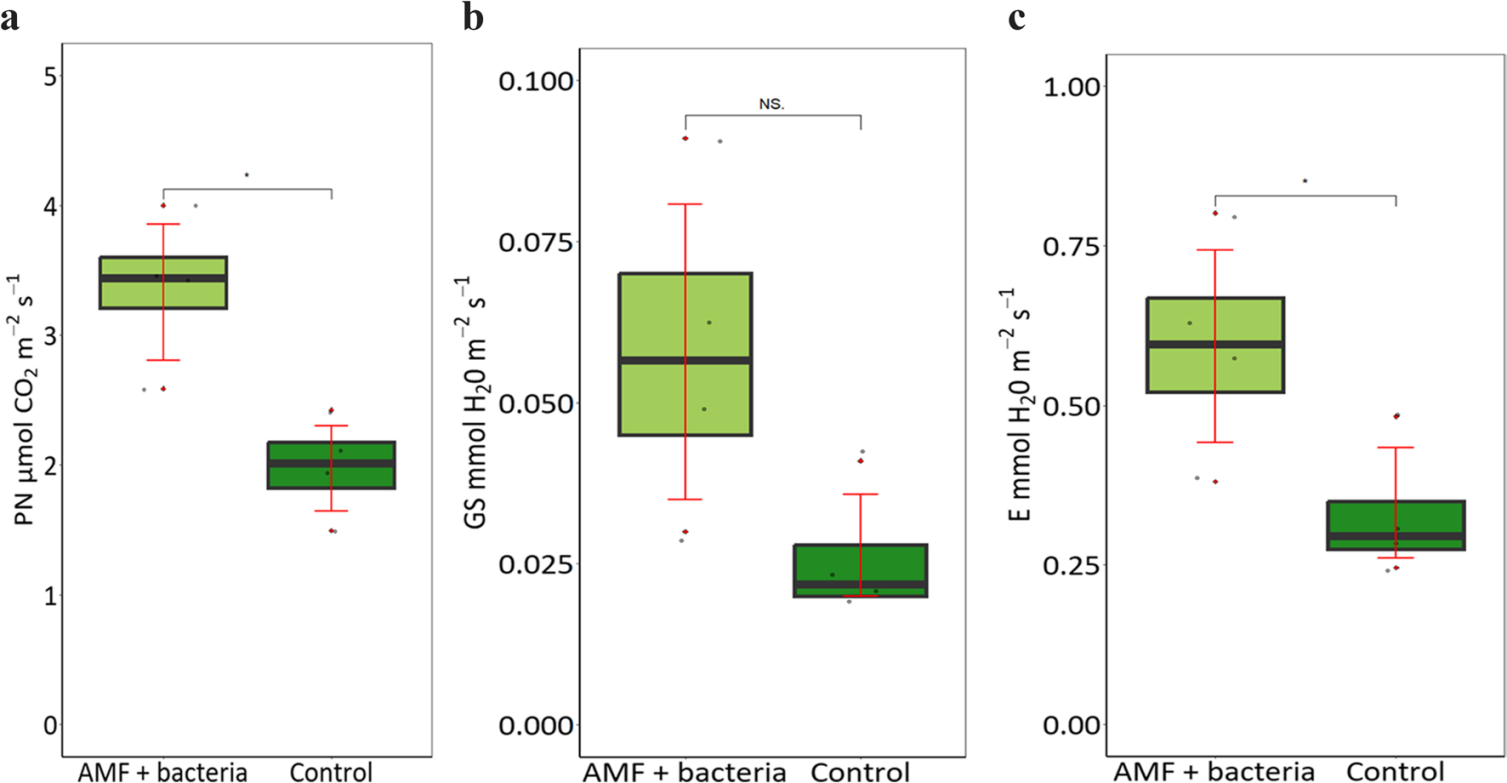
Physiological parameters of plants from semi-industrial scale experiment measured 2 weeks after a temporary water shortage: **A** photosynthetic rate (PN, mmol CO_2_·m^−2^·s^−1^), **b** stomatal conductance (GS, mmol H_2_O·m^−2^·s^−1^) and **c** transpiration rate (E, mmol H_2_O·m^−2^·s.^−1^) of red raspberry co-inoculated with AMF and AMF spore-associated bacteria. Statistically significant differences between inoculated and non-inoculated plants are indicated by asterisk (*t*-test, *P* ≤ 0.05, *N* = 5)

Two weeks after a temporary water shortage, gas exchange in plant leaves was examined to verify if inoculation improved C assimilation. Photosynthetic rate, stomatal conductance of H_2_O and transpiration rate were improved in inoculated plants. CO_2_ assimilation (photosynthetic rate) was increased by 70%, stomatal conductance was improved over two-fold and the rate of transpiration was increased by 80% (Fig. 3).

## Discussion

Biotization with microorganisms may result in changes in plant development and physiology facilitating adaptation to the environment [20]. The appropriate selection of microorganisms for this purpose is of utmost importance. The development of sustainable horticulture requires reducing usage of chemical pesticides and fertilisers and improvement of plant productivity. One symbiotic microorganism may not fully cover the needs that crops require for optimal growth [21]. On the other hand, multi-microbial inocula may not be effective for different crop species and even for different cultivars of the same species [24, 34]. Therefore, better understanding of the compatibility between symbiotic microorganisms and plants is required for targeted biotization of crop plants [55]. In the vast majority of previously published studies, plants were inoculated with single AMF species [49], and their response varied from inhibition to activation of plant growth, depending on AMF species. The synergistic effect of AMF and other factors (soil organic matter, insect pollination, nutrient availability) on the production of raspberry has also been examined on a few occasions [12, 13]. Gianinazzi et al. [21] showed that synergistic effect of AMF and soil-borne strain of *Paenibacillus* protected tomato against pathogenic *Phytophthora parasitica.* However, the synergistic effect of AMF and AMF spore-associated bacteria has not been investigated.

We isolated over fifty strains of bacteria from spores of AMF. It has to be emphasised that these bacteria most probably resided either within the cell wall or on the outside of the spore, thus being accessible for isolation and cultivation. Up until now only a limited number of reports describe the community structure of bacteria residing inside spores. According to Bianciotto et al. [6] and Naito et al. [33], only two bacterial taxa: ‘*Candidatus* Glomeribacter gigasporarum’ and ‘*Candidatus* Moeniiplasma glomeromycotorum’ were shown to inhabit spore interior However, Lastovetsky et al. [27], in a recently published paper, have shown a larger diversity of bacteria inhabiting AMF spores. Only some of them showed plant growth-promoting properties, and the best strains were selected for plant inoculation. Six bacterial strains, which were selected, belonged to *Paenibacillus* genus. Representatives of this genus have been previously shown to associate with AMF spores [1, 29] and additionally to inhibit the growth of soil-borne pathogenic fungi [16]. The results of our laboratory experiment showed that bacteria isolated from AMF spores alone did not improve plant growth or vitality. Co-culture with AMF was required for plant growth activation. AMF alone had a beneficial impact on plant yield, whereas supplementation with AMF and bacteria from AMF spores had a synergistic effect on raspberry growth. It was assumed that the bacteria may improve root colonisation by AMF,plant biomass yield was often related with high root colonisation by AMF [14, 45]. Other reports, however, did not show such a relationship [19, 30]. Here, supplementation with bacteria did not affect root colonisation by AMF (data not shown). It should be noted though that the majority of mycorrhizal colonisation parameters reached over 90% in AMF inoculated plants not supplemented with bacteria.

In the tunnel experiment, the beneficial effect of co-inoculation with AMF and bacteria on raspberry growth and vitality was verified positively. Plant growth parameters (high, fresh weight, dry weight) were improved by 28–76%, and selected parameters of chlorophyll *a* fluorescence were increased. These results indicate that biotization of raspberry with AMF and AMF spore-associated bacteria may be an alternative to conventional methods in large-scale raspberry production.

One of the main barriers to crop production is drought. Therefore, methods to increase plant resistance against drought are of particular interest for farmers and scientists. Arbuscular mycorrhizal fungi, endophytic fungi and endophytic bacteria have been documented as biological agents capable of improving drought resistance in crop plants [2, 35, 37, 47, 57]. Plant inoculation with symbiotic microorganisms often improves the absorptive surface of the roots and the activity of stress protective agents, osmoregulation, and antioxidant capacity [42, 43]. Thus, we investigated the effect of temporary water shortage (7 days) on basic physiological parameters of biotized raspberry. Two weeks after the treatment, the tested physiological parameters of inoculated plants were clearly improved. Plant inoculation with AMF and spore-associated bacteria improved stomatal conductance. Increased aperture of stomata promotes more efficient diffusion of CO_2_ and H_2_O; hence, in our study, a significant increase in transpiration rate was observed in inoculated plants. Better diffusion of CO_2_ through the stomata allows its efficient distribution in the stroma of chloroplasts increasing the intercellular carbon dioxide concentration and thus reducing the likelihood of photorespiration [26]. Inoculated plants were characterised by increased concentrations of intercellular CO_2_ (unpublished data). At the same time, we observed an increased proportion of active reaction centres and more efficient transport of electrons out of PSII in plants supplemented with the tested inoculum. Such functional remodelling of the photosynthetic apparatus increases the efficiency of capturing incoming radiation and the efficiency of linear transport of electrons [50]. The observed changes resulted in a significant increase in the rate of carbon dioxide assimilation in inoculated plants.

In conclusion, our results indicate that biotization of raspberry with arbuscular mycorrhizal fungi and selected bacteria isolated from spores significantly improved plant growth and biometric photosynthetic activity. Moreover, physiological performance of inoculated plants was improved compared to non-inoculated plants after temporary water shortage, suggesting improved resistance to drought. This shows that biotization with AMF and bacteria isolated from spores has potential application in raspberry production. This is particularly important due to the increasing demand for horticultural methods that rely on plant-microorganisms interaction [20].

## Data Availability

No datasets were generated or analysed during the current study.
